# A Single-Center Surgical Experience With the Reverse Sural Artery Flap as a Reliable Solution for Lower Leg Soft Tissue Defects, With Minimum Two-Year Follow-Up

**DOI:** 10.7759/cureus.16574

**Published:** 2021-07-22

**Authors:** Efstratios D Athanaselis, Apostolos Fyllos, Aristeidis H Zibis, Theofilos Karachalios, Michael Hantes, Zoe Dailiana, Konstantinos Malizos, Sokratis Varitimidis

**Affiliations:** 1 Orthopaedics and Musculoskeletal Trauma, University Hospital of Larissa, Larissa, GRC; 2 Anatomy, School of Health Sciences, University of Thessaly, Larissa, GRC; 3 Orthopedics and Musculoskeletal Trauma, University Hospital of Larissa, Larissa, GRC

**Keywords:** soft tissue defect, reconstruction, complication, diabetic foot ulcers, cutaneous nerve, artery, pedicled flap

## Abstract

Aim: Small soft tissue defects of the distal tibia and hindfoot resulting from traumatic, operative, or neoplastic conditions and chronic ulcers can be successfully dealt with the use of the reverse sural artery flap (RSAF). This study aims to describe a single center’s results and familiarity with this technique over a 15-year period of time.

Material and methods: We retrospectively reviewed the clinical files of patients who were consecutively treated with RSAF and regularly followed up between January 1, 2004 and December 31, 2018, with a minimum postoperative follow-up period of two years. Patient demographics and comorbidities, location of the defect, performing surgeon, mean operation time, flap pedicle width, mean size of the defect, days of hospitalization following the operation, healing flap rate, and complications were recorded.

Results: The sample consisted of 30 adult patients (25 men, 5 women), with a mean age of 51.07 years (16-80 years, SD 18.61). The mean operation time was 99.03 min (range 83-131, SD 10.57), and the mean size of the defect was 11.11 cm^2^ (range 6.1-19.4, SD 3.22). Successful flap rate (complete healing and coverage of the defect, with or without additional minor intervention) was 83.3% (25/30). Among successfully healed flaps, six patients with partial necrosis of the dermis were treated by an additional split-thickness skin graft. Five flaps failed to heal. Deep infection was present in two patients, leading to flap failure and reoperation. Serious venous congestion resulting in flap ischemia occurred in three cases. Circumferential keloid formation (not affecting successful outcome) was present in seven cases. Flap thickness approximated to normal within six months. All donor sites healed well (either by a split-thickness cutaneous flap or by immediate wound closure). Light paresthesia on the lateral border of the leg and foot disappeared within six months.

Conclusions: A single-center experience with the RSAF has yielded satisfactory clinical outcomes, and the long-term tackle with the difficult reconstruction conditions around the ankle, has led to valuable advice on surgical technique and postoperative protocol, based on an anatomical basis.

## Introduction

The distal third of the leg is an anatomic region where the use of fasciocutaneous flaps is often imposed by the need for soft tissue defects’ coverage. Restricted availability of skin for primary closure after injuries, operative interventions, and complications as well as chronic ulcers, require flap coverage of numerous tendons, vessels, and exposed bone. If these parameters are not taken into proper consideration, the functional and aesthetic results can be poor.

Reverse sural artery flap (RSAF) is among various flaps proposed for the reconstruction of the distal leg, ankle, and foot defects, such as the lateral supra malleolar flap, peroneus brevis and extensor digitorum brevis muscle flaps, and crossed leg flaps. RSAF is a reliable, pedicled, local fasciocutaneous flap from the sural angiosome, suitable for heel and hindfoot defects, contained in the area of the posterior calf between the popliteal fossa and midportion of the leg [1]. It is centered over the middle raphe between the medial and lateral heads of the gastrocnemius muscle and can be raised as fasciocutaneous, adipofascial, and myocutaneous. It can be used to cover exposed vessels, bones, tendons, and internal fixation hardware [2]. It has a wide arc of rotation, which allows for coverage from the lower half of the leg up to the metatarsophalangeal joints on the dorsal aspect and base of the metatarsal bones on the plantar aspect of the foot [3,4]. Compared with the distally based peroneus brevis and the extensor digitorum brevis muscle flaps, the RSAF can be applied in larger defects [5,6]. It is an extremely versatile option, both proximally and distally, and has been used to cover even very large defects (areas as large as approximately 17 × 16 cm^2^ to 20 × 15 cm^2^) [3,4]. Immobilization and difficult positioning are avoided unlike the cross-leg flaps [7]. The lateral supra malleolar flap despite its technical ease has fewer indication options sizewise and results in a larger insensate area of the foot [8]. Therefore, RSAF is popular among surgeons for its reliability, relative technical ease, lack of dependency on microsurgery, preservation of the major arterial supply to the lower limb, and relative economic liability to the patient and the health care infrastructure [2,3]. It is also the preferred method of treatment, when necessary, in our institution, including surgeons not specialized in microsurgery.

The main indications for the fasciocutaneous RSAF are coverage of chronic skin ulcers, traumatic lesions (mainly secondary to open fractures), chronic osteomyelitis, oncological resections, wound dehiscence at the posterior aspect of the heel and of the Achilles tendon, the lateral and anterior aspect of the ankle, the back of the foot, the lateral aspect of the hindfoot, and the lower third of the leg [9-11]. Other indications, such as full heel coverage and medial aspect of the distal third of the leg, are considered relative because of the small distance to the rotational point, which may affect the vascular pedicle at attempts to reach these regions, compromising the flap [9-11]. Defects of the heel (28.2%), foot (14.4%), or ankle (25.8%) represent the majority of locations for RSA flap use, while it is most commonly used for defects with mean flap dimensions calculated to be 8.4 × 5.7 cm^2^. Trauma has been the most common indication, followed by ulcers and open fractures [12].

The aim of this study is to present the long-term clinical results and share the surgical technique and experience of the Orthopaedics and Musculoskeletal Trauma Unit of the University Hospital of Larissa with the RSAF, over a 15-year period of time.

## Materials and methods

**Figure 1:**
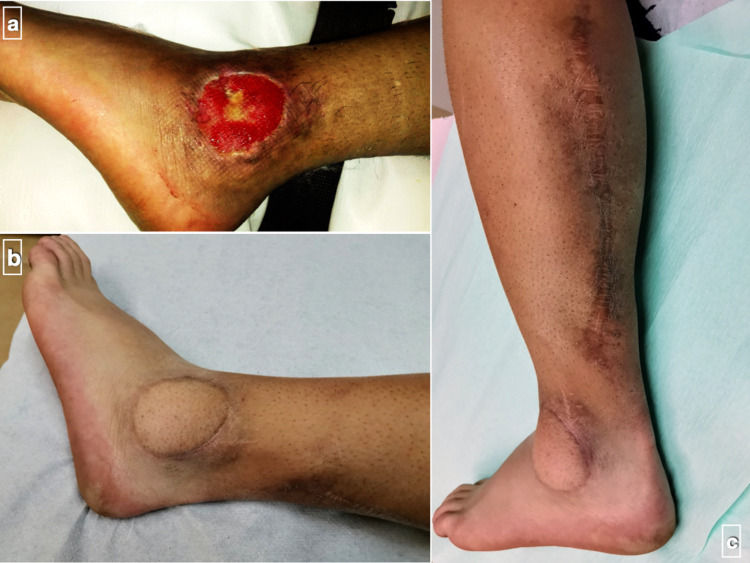
Successful use of reverse sural artery flap (a) The initial skin defect created by a soft tissue excision over the lateral malleolus of a male patient in his early 20s. (b and c) The long-term (two years postoperatively) result after coverage with reverse sural artery flap.

We retrospectively reviewed the clinical files of patients that were consecutively operated on for soft tissue defects using RSAF as a primary procedure between January 1, 2004 and December 31, 2018. Inclusion criteria were patients over the age of 16 years, soft tissue defects over the lower extremity requiring reconstruction due to acute or chronic trauma, tumor excision, and chronic osteomyelitis, and a minimum postoperative follow-up period of two years (Figure 1). Patients with vascular injury or diagnosed peripheral arterial disease were excluded from the study. Data concerning patient demographics, location and cause of defect, mean operation time, mean size of the defect, postoperative protocol, healing flap rate, and complications were recorded. Comorbidities and details of patients with flap failure are also presented.

Surgical technique

The position of the patient depends on where the defect is located. Our experience suggests the prone position to be the most convenient position for the performing surgeon for raising the RSAF, and this is certainly used for posterior defects on the heel or over the Achilles tendon. In case the defect is on the anterior ankle and foot, lateral decubitus position with the patient on the contralateral side is more suitable. However, it is important to remember that when the patient is at a lateral decubitus position, the topographic anatomy of the leg may be altered due to calf distortion.

**Figure 2:**
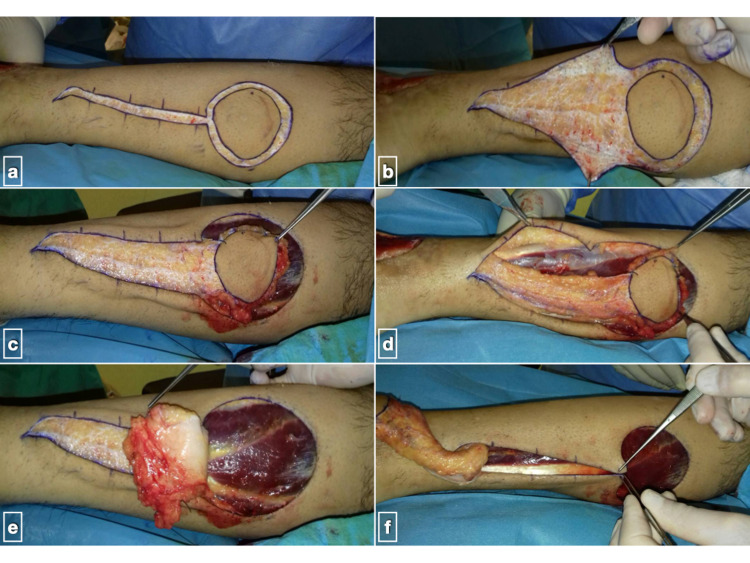
Case from the previous figure (a) Skin incisions for an island reverse sural artery flap; (b) careful subdermal dissection of flap pedicle; (c and d) separation of fasciocutaneous flap; (e) flap is raised together with deep fascia which functions as a firm basis; (f) flap is placed at the recipient site sutured loosely and the donor site is checked for primary closure.

The flap is designed according to the size and shape of the defect area, taking into account the distance from the defect area to the point of the pedicle’s rotation (Figure 2). Both the skin island and pedicle are elevated from proximal to distal in the subfascial plane to the level of the chosen pivot point (Figure 2). A pre- or peri-operative Doppler or angiography study to evaluate vascularity of the flap is not performed as a routine. However, flap pedicle width has been standardized to be not less than 4 cm and its pivot point must be at least 5 cm proximal to the lateral malleolus. The flap is then transferred to the recipient site leaving the pedicle over the intervening intact skin unless soft tissue laxity at the area allows subcutaneous tunneling. In both cases, the pedicle must be rested without tension or pressure. The donor site is treated with immediate wound closure (Figure 2) or covered by a split-thickness skin graft. In cases of flap venous congestion, the patient’s hospital stay is extended for close monitoring and appropriate therapeutic measures are taken, such as limb elevation, skin scarifications, and removal of potentially constricting skin sutures.

Postoperative period

**Figure 3:**
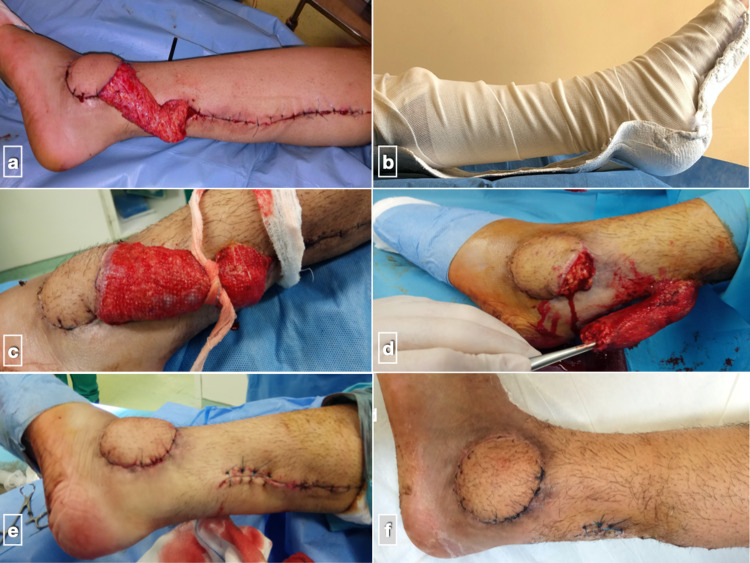
Case from previous figures (a) Immediate postoperative result, after tourniquet release; (b) modification of standard back slab, in order to avoid external pressure to pedicle and flap when patients rest their leg; (c) flap’s neo-vascularization is tested three weeks later by strangling the pedicle; (d) if adequate blood supply is maintained, the flap is separated from the pedicle; (e) the pedicle is removed and remaining skin incisions are closed; (f) uneventful flap healing at four weeks postoperatively.

Leg immobilization in the early postoperative period is of utmost importance. The flap should be free of pressure and other mechanical forces. Therefore, a modified back slab is used (Figure 3), and the limb is elevated for at least two weeks to prevent venous congestion. Thromboembolic prophylaxis is administered for one month as per institution protocol (low-molecular-weight heparin + low dose aspirin). Antibiotic therapy (cefazolin) is maintained routinely for 48 hours postoperatively. The postoperative protocol consists of hospitalization for four to five days followed by a routine check on an outpatient basis every second day up to 8-10 days. The flap is separated three weeks later as a day case surgery, after confirming autonomous vascularity by blocking the pedicle’s arterial flow (Figure 3). No flap separation is obviously needed in case of subcutaneous transfer. Follow-up examination continued at one, two, three, six months, and yearly.

## Results

Thirty patients were found to fulfill all criteria mentioned above (Table 1). The majority of patients were men (25/30). The mean sample age was 51.08 years (range 16-80, SD 18.61). Localization of the primary defect varied. In the majority of patients, the lateral malleolus was the recipient site (10/30), followed by the posterior lower leg (Achilles tendon) (7/30), the anterior distal leg (6/30), the medial malleolus (4/30), and the heel (3/30). The mean area of the defect was 11.11 cm^2^, ranging from 6.1 to 19.4 cm^2^ (SD 3.22). The meantime of operation (from tourniquet inflation to dressing application) was 99.03 min (range 83-131, SD 10.57). Two flaps were subcutaneous. The remaining 28 patients had their flaps separated. Twenty-five flaps were considered successful.

**Table 1:** Descriptive statistics and results of eligible patients

Data	Results
Sample size (male/female)	30 patients (25 male/5 female)
Mean age in years (range, SD)	51.07 (16-80, 18.61)
Recipient site	Lateral malleolus (malignancy, inability for skin closure following osteosynthesis with exposed bone) 10/30
	Posterior distal leg (exposed Achilles tendon following open trauma/burn or postoperatively) 7/30
	Anterior distal leg (open trauma/burn or postoperatively following osteosynthesis, with exposed anterior tibialis tendon) 6/30
	Medial malleolus (inability for skin closure following osteosynthesis with exposed bone) 4/30
	Heel (pressure ulcer, malignancy, with exposed bone) 3/30
Mean operation time in minutes (range, SD)	99.03 (83-131, 10.57)
Mean defect size in cm^2^ (range, SD)	11.11 (6.1-19.4, 3.22)
Successful flap (rate)	25/30 (83.3%)

**Figure 4:**
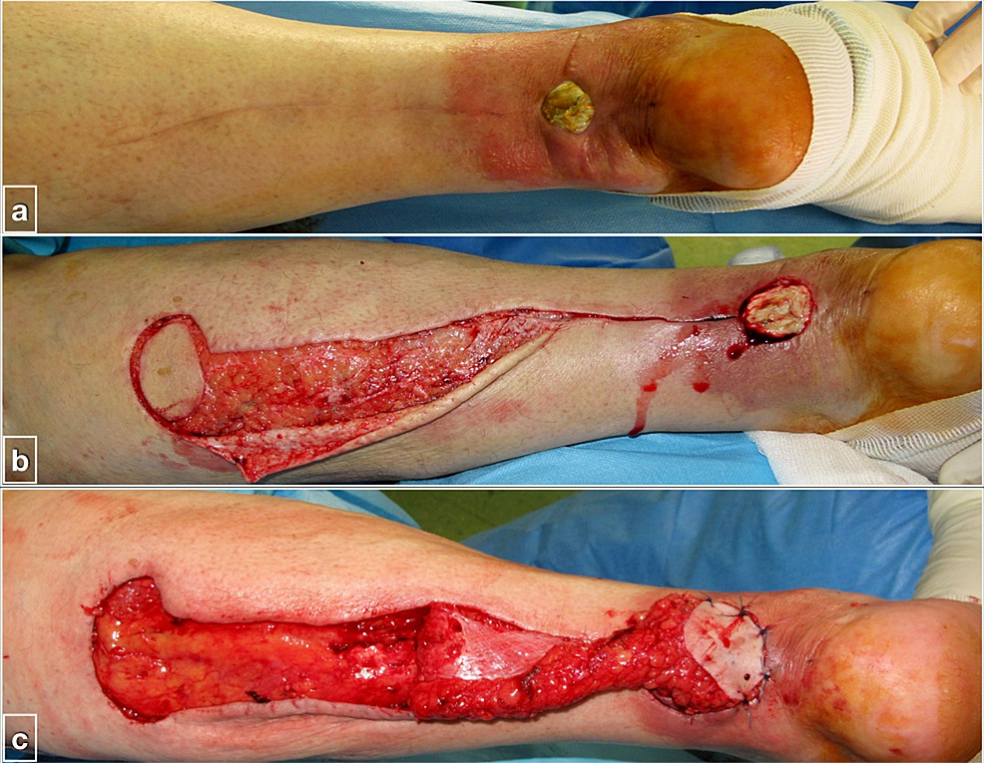
Complications following use of reverse sural artery flap (a) Skin defect on the posterior lower leg of a female patient in her early 70s with type II diabetes mellitus, multiple steroid injections at the area for tendinitis, re-rupture of repaired Achilles tendon, and surgical wound breakage; (b and c) wound debridement, reverse sural artery flap preparation, and coverage of the defect.

**Figure 5:**
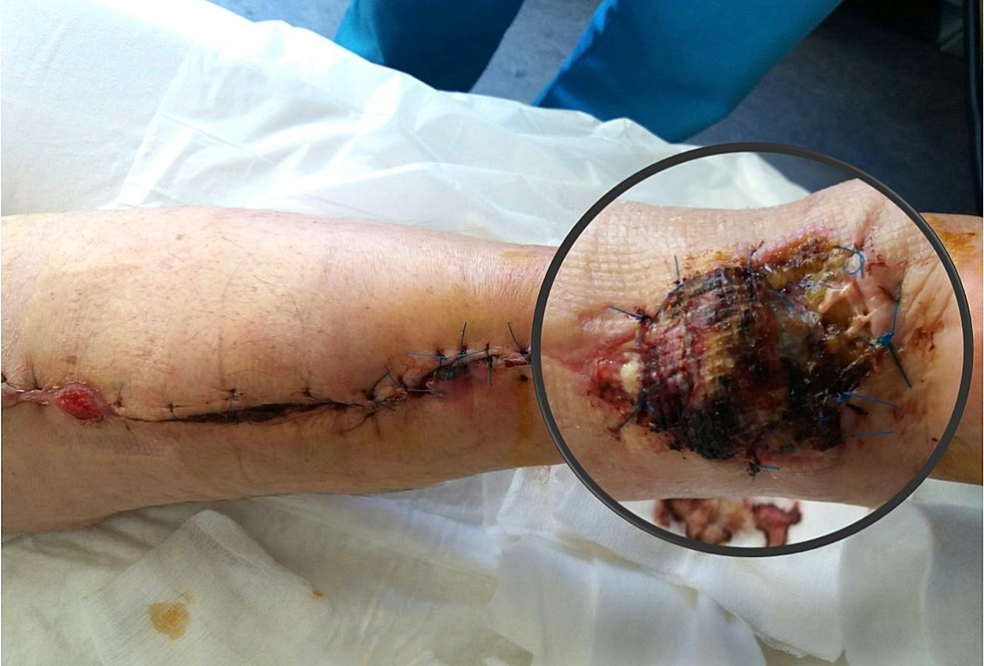
Flap failure due to infection by Proteus mirabilis The patient was secondarily treated with a free anterolateral thigh fascia lata flap to replace Achilles tendon defect in a Plastic Surgery Unit.

Complete flap failure was observed in five patients (16.7%). Two were observed in patients with comorbidities. The first was a 70-year-old female patient with type II diabetes mellitus with a posterior leg defect following Achilles tendon repair (owing to multiple steroid injections), re-rupture, and wound breakage. The flap was complicated by deep infection by *Proteus mirabilis* and ultimately failed (Figures 4 and 5). The other was a male patient in his mid-thirties, a smoker, suffering from paraplegia and mild venous insufficiency, with a chronic ulcer over the medial malleolus. The flap was complicated by venous congestion and deep infection and ultimately failed. The other three patients with flap failure due to venous congestion were relatively young with no comorbidities.

Out of 25 flaps considered healed and successful, partial necrosis of the dermis was treated by an additional split-thickness skin graft after surgical debridement in six patients. Circumferential keloid formation was present in seven cases. Flap thickness approximated to normal within 8-12 months. Therefore, no debulking procedures were deemed necessary. The range of motion of the ankle and foot was within normal values, as recorded at six months. There were no patient complaints or complicated insensitivity (i.e., ulcer formation) related to the sacrifice of the sural nerve. Light paresthesia on the lateral border of leg and foot disappeared after two to three months. No donor site complications were recorded.

## Discussion

Clinical results of RSAF were quite favorable in this series of patients from a single center. We considered a flap as "successful" when complete healing was achieved, with complete coverage of the defect, with or without additional minor intervention (such as surgical debridement and split-thickness skin graft). Successfully healed flaps consisted of 83.3% of all flaps, with a mean size of defects measured to be approximately 11 cm^2^. The most common site requiring coverage was the lateral malleolus (33.3% of patients), followed by the skin over the Achilles tendon (23.3%). No debulking procedures were required and no donor site morbidity was observed. In the following paragraphs, several correlations have been made between previously published results and our experience with this technique.

Anatomic basis of surgical technique

The RSAF is based on the anatomical concept of developing a neurocutaneous island flap based on the cutaneous branches of the vascular axis around a superficial sensory nerve, without sacrificing a major artery of the lower limb [13]. Apart from the anterograde blood supply from the sural superficial arteries, there are at least four sources of retrograde blood supply: fasciocutaneous perforators from the peroneal artery, fasciocutaneous perforators from the posterior tibial artery, venocutaneous perforators from the lesser saphenous vein, and neurocutaneous perforators from the sural nerve [2]. These form chain-linked vascular plexuses around the nerve and vein contained in the pedicle, by connecting with each other and with the anastomosis of the vascular networks from the superficial fascia, deep fascia, and subdermis [14,15]. The distal-most fasciocutaneous perforator from the peroneal artery on which the pivot point is chosen has been found to be situated at an average of 5.3 cm (range 2-9 cm, SD 2.1) and 5 cm (SD 1.3 cm) above the distal tip of the lateral malleolus according to two cadaveric studies. These vessels are anastomosed directly with the superficial sural arteries that form a dense arterial network known as the sural angiosome [16,17]. The lateral calcaneal artery and the lateral malleolus artery (the two terminal branches of the peroneal artery) also spawn cutaneous perforators, located approximately 1 and 3 cm proximal to the tip of the lateral malleolus, respectively [17]. Schaverien and Saint-Cyr examined peroneal perforators and found that are randomly distributed and can be found at any level, making careful dissection mandatory, in order to identify a proper vascular source for the flap [18]. Most authors, however, advocate the use of pre-operative Doppler ultrasound to ensure adequate flap vascularity [2,19-22]. In our practice, the flap pedicle must be at least 4 cm wide in order to secure adequate arterial supply and venous drainage. The deep fascia, though it does not contribute to the vascular supply, serves to strengthen the flap and should be used in island skin flaps and adipofascial flaps [16].

Surgical anatomy of the sural nerve 

The main disadvantage of the RSAF technique is the sacrifice of the sural nerve, resulting in sensory loss over the lateral foot. The medial sural nerve courses sub-facially alongside the tibial nerve while passing between the two heads of the gastrocnemius muscle. The lateral sural nerve runs across the lateral gastrocnemius belly also in the subfascial plane. As both nerves travel distally, they both enter the subcutaneous plane by piercing the muscle fascia and merge 1-2 cm distal to the subcutaneous entry point to form the common sural nerve (adjacent to the short saphenous vein) at a mean distance of 13.6-14.5 cm (range 9.4-18.2 cm) proximal to the lateral malleolus, according to two anatomical studies [15,23]. In another study, the mean distance from the heel was measured to be 17.1 cm (range 13.0-21.8 cm) [16]. Furthermore, there is approximately 25% possibility of anatomical variation of the course of the sural nerve, either as two separate medial and lateral sural cutaneous nerves or as a “diminishing” pattern of the lateral sural cutaneous nerve [23,24]. The technique was first described by Donski and Fogdestam and later championed by Masquelet et al., as a distally based neuro-skin flap, supplied by the vascular axis of the sural nerve [13,25]. More recently, it has been found that despite the fact that the sural nerve contributes to the vascular network, its inclusion in the flap does not appear to increase the arterial blood supply. However, it allows a more distal pivot point, increasing the arc of rotation of the sural flap [16]. Techniques of RSA flap harvesting with preservation of whole or part of the sural nerve have been published, requiring microsurgical familiarization and equipment, increasing operative time [15,23]. However, minimal complications or complaints regarding foot sensation appear to be reported when the “traditional” technique is used [4,11].

Venous anatomy leading to surgical technique modification 

The distally based sural artery flap has two venous drainage systems: the short saphenous vein and the vena comitantes of the peroneal perforator. The blood flow in the short saphenous vein is in reverse fashion while that in the vena comitantes is anterograde. Venous blood from the flap is primarily drained to the lesser saphenous vein by reverse flow. Valves in the deep venous system prevent this reverse flow and could lead to venous congestion and flap failure, which is considered the most serious complication of this technique [26]. RSAF is distally based and the pedicle needs to be folded and rotated almost 180°, which can cause venous congestion due to the new position of the venous valves and the kinking of the vessels [27]. However, the venae comitantes and their communicating branches allow backflow. Pedicle width of a minimum of 4 cm has been found to greatly improve venous outflow, not only because it includes a sufficient venous net, but also by avoiding acute angle kinking [19].

Factors affecting healing rate

Flap necrosis is more often encountered in patients with large flap designs and comorbidities. Based on meta-analyses, the incidence of flap failure ranges from 3.2% to 8%, although surgical technique, sample demographics, and indications may vary across included studies [2,10,12]. Mean flap size has been calculated to approximate 40 cm^2^, and larger flap sizes appear to affect outcome and complications rate, but the flap aspect ratio does not [10,12]. Recent research suggests that primary flap healing might depend on its location above or below the ankle joint. The flap success rate was significantly higher (65%) in patients with injuries at or proximal to the level of the ankle joint, compared with only 42% in patients with injuries distal to the level of the ankle joint [3]. Baumeister et al. found the age of more than 40 years to be a significant risk factor for complications regardless of comorbidities. In the same study, patients without medical problems showed a necrosis rate of 11%, and patients with systemic diseases other than diabetes mellitus, venous insufficiency, and peripheral arterial disease showed a necrosis rate of 33%. Patients with one or more of those three diseases showed a necrosis rate of 60% [28]. More recently, de Blacam et al. exploring the same research question, concluded that severe venous insufficiency and increasing age were independent risk factors for the development of complications. However, they also found that peripheral vascular disease, smoking, and diabetes did not correlate with an unfavorable outcome or increased complication rate [12].

Perioperative and postoperative considerations

In our study, the mean age of patients (51.08 years) is slightly higher, compared to the usual patient profile according to published literature [11,12,19]. Mean operation time is seldom reported. In a recently published large series, it was measured to be approximately 121 min, significantly longer than our series (99.03 min, range 83-131 min). However, the mean size of defects in that study was also significantly larger [11]. Postoperatively, Severo et al. suggest that apart from smoking discouragement, special dietary caution should be taken against food that can cause vasoconstriction, such as coffee, guaraná, chocolate, chimarrão (South Brazilian traditional tea), stuffed wafer, tea, and other xanthine-rich foods should be avoided for at least one month [9]. Other authors have commented on the importance of avoiding flap external pressure and limb elevation during the immediate postoperative period [2,9,19]. Rothenberger et al. studied optimal leg position (on the fifth postoperative day) and observed that the best blood flow and lowest hemoglobin concentration was in the 45° upward position. They assumed that this reduction in hemoglobin concentration was associated with venous congestion reduction rather than reduced blood perfusion. Nevertheless, these differences were significant compared to the 45° downward leg position, but not when compared to the horizontal position of the limb [29].

Complications

The RSA flap has an overall favorable complication profile since 82% of flaps have been reported to heal without any flap-related complications [2]. Complication rates do not appear to be influenced by the learning curve and a very weak correlation between surgeon experience and the incidence of failed reconstructions has been established [9,19]. De Blacam et al. observed a steady increase in the use of the sural flap in literature, characterizing it as a trend away from the more complex microsurgical reconstruction of lower limb wounds that may have a higher economic impact on healthcare [12]. Overall, flap survival seems to be highly dependent on meticulous operative technique and modifications since its original description enhances venous outflow [2,19,30]. The success rate (83.3%) in our study could be perceived as high, considering that not all surgeons performing RSAF surgery were specialized in microsurgery or plastic surgery. However, postoperative complication probability must be individualized, and the patient expectations appropriately adjusted.

Limitations

This study has a number of limitations: no biometrics or other habits or comorbidities were available and consequently not analyzed as possible factors affecting the outcome. Information on how long the wound had been open and colonized before reconstruction is not reported. There are inherent methodology flaws and bias due to the single-center and retrospective nature of this research. For example, no subjective scores were recorded during the outpatient follow-up visits, and a good result is hypothesized by the lack of recorded complications or readmissions. Finally, the lack of routine use of Doppler perioperatively creates a limitation in the surgical technique, although this practice caused no perioperative problems.

## Conclusions

The sural flap is an effective and widely used solution for lower leg skin defects. As a local, rotational flap, it can be applied in post-traumatic, post-operative skin lesions and chronic ulcers around the ankle and the hindfoot that need coverage. Even though microsurgical familiarity is desirable, it is not necessary and this flap may be useful in healthcare settings where microsurgical methods are not available. Nevertheless, RSAF demands meticulous surgical techniques. Our long-term tackle with the difficult reconstruction conditions around the ankle has led to valuable advice on surgical technique and postoperative protocol, based on an anatomical basis. The adequate thickness of the pedicle can reduce flap failure which, in most cases, is caused by impaired venous drainage.
